# Identification of hub genes and drug candidates for NF2-related vestibular schwannoma by bioinformatics tools

**DOI:** 10.1097/MD.0000000000036696

**Published:** 2023-12-15

**Authors:** Jiasheng Yuan, Yanpeng Fu, Yuehui Liu

**Affiliations:** a Department of Otorhinolaryngology Head and Neck Surgery, The Second Affiliated Hospital, Jiangxi Medical College, Nanchang University, Nanchang, Jiangxi, China.

**Keywords:** bioinformatic analyses, molecular mechanism, NF2, vestibular schwannomas

## Abstract

Neurofibromatosis type 2 (NF2)-related vestibular schwannoma (NF2-VS) is a rare genetic disorder that results in bilateral acoustic neuromas. However, the exact pathogenesis of the disease is still unclear. This study aims to use bioinformatics analyses to identify potential hub genes and therapeutic. We retrieved the mRNA expression profiles (GSE108524 and GSE141801) of NF2-VS from the database, and selected the leading 25% genes with the most variance across samples for weighted correlation network analysis. Subsequently, we conducted gene ontology term and Kyoto Encyclopedia of Genes and Genomes signaling network enrichment analyses. The STRING database was employed for protein-protein interaction (PPI) axis construction. The mRNA-miRNA modulatory network was generated via the miRTarBase database. Differentially expressed genes (DEGs) were identified via the R package “limma” in both datasets, and hub genes were screened via intersection of common DEGs, candidate hub genes from the PPI axis, and candidate hub genes from the key module. Finally, common DEGs were uploaded onto the connectivity map database to determine drug candidates. Based on our observations, the blue module exhibited the most significant relation to NF2-VS, and it included the NF2 gene. Using enrichment analysis, we demonstrated that the blue modules were intricately linked to modulations of cell proliferation, migration, adhesion, junction, and actin skeleton. Overall, 356 common DEGs were screened in both datasets, and 33 genes carrying a degree > 15 were chosen as candidate hub genes in the PPI axis. Subsequently, 4 genes, namely, GLUL, CAV1, MYH11, and CCND1 were recognized as real hub genes. In addition, 10 drugs with enrichment scores < −0.7 were identified as drug candidates. Our conclusions offered a novel insight into the potential underlying mechanisms behind NF2-VS. These findings may facilitate the identification of novel therapeutic targets in the future.

## 1. Introduction

Vestibular schwannomas (VSs) are benign neoplasms of Schwann cell-derived vestibulocochlear nerves.^[1,2]^ Approximately 90% of VSs patients experience progressive hearing loss, along with other symptoms like tinnitus and dizziness. The lifetime VS risk is estimated to be at 1 in 1000.^[3]^ Most VSs appear sporadically, and are located primarily within the cerebellopontine angle. Approximately 5% of VS cases involve the neurofibromatosis type 2 (NF2), present bilaterally, and have a genetic predisposition.^[4]^ Bilateral VS is a typical Nf2-related vestibular schwannoma (NF2-VS) lesion. The NF2-VS is a rare genetic disease brought on by the inactivation of the NF2 gene on chromosome 22q12.2.^[4,5]^ The NF2 gene product, merlin, is generally expressed at high levels in Schwann and nerve cells among adult individuals. Given that merlin contributes heavily to cell-to-cell proliferation and adhesion, merlin deficiency can potentially impair contact growth inhibition function.^[6]^ Till date, there are no reports on the exact mechanism of NF2-VS pathogenesis.

Gene expression profiling is a robust approach for identifying essential pathological mechanisms,^[7]^ and one form of gene profiling, microarray-based gene expression profiling, is frequently used to screen for differentially expressed genes (DEGs) or novel biomarkers in numerous human diseases.^[7]^ The Gene Expression Omnibus database contains certain VS gene expression profiles,^[8]^ the integration and reanalysis of which may provide valuable insight into the pathogenetic pathways of NF2-VS. Weighted correlation network analysis (WGCNA) is a bioinformatics tool that describes association profiles between different genes.^[9]^ WGCNA incorporates genetic and clinical information to precisely identify relevance of certain genes among various samples. Herein, we, for the first time, screened for candidate NF2-VS-related hub genes, and identified several efficacious therapeutic drugs for NF2-VS using bioinformatics analysis.

## 2. Materials and methods

### 2.1. Data accumulation

Publicly available NF2-VS mRNA datasets were downloaded from the Gene Expression Omnibus website (https://www.ncbi.nlm.nih.gov/geo/). GSE108524, compiled using the GPL17586 platform of the Affymetrix GeneChip Human Genome HTA_2_0 Array, contains 31 human samples stratified into 3 categories: 17 NF2-VSs, 10 sporadic VSs, and 4 normal nerves. In contrast, GSE141801, compiled using the GPL13667 Affymetrix Human Genome U219 Array, contains samples from 9 recurrent irradiated VSs (5 sporadic, 1 cystic, and 3 NF2-VSs), 36 sporadic VSs, 13 NF2-VSs, and 7 control vestibular nerve tissues. Only the NF2-VSs and control nerves from both datasets were analyzed in this study. Data standardization was performed using the R 4.0.3.

### 2.2. Construction of a co-expression network

“WGCNA,”^[9]^ an R package, was used to conduct WGCNA. The “pickSoftThreshold” tool was employed for the soft-thresholding power measurement. Subsequently, a co-expression network module was developed using the “one-step network construction and module detection method,” and the minimum gene quantity within the modules was set to 30.

### 2.3. Selection of the NF2-VS-related module

For the determination of major components of various modules, we employed module eigengenes (MEs), which utilize the first principal component of module expressions. We also evaluated the association between MEs and patient clinicophysiological information, and generated heat maps.

### 2.4. Functional enrichment analysis (FEA)

To elucidate the roles of NF2-VS-related genes within the modules, we performed FEA of the strongly associated modules using the clusterProfiler package of the R software.^[10]^ *P* value adjustment was done via the Holm–Bonferroni technique. The adjusted *P* and the *q* value thresholds were set to 0.05.

### 2.5. Generation of a protein-protein interaction (PPI) axis

The key module genes were uploaded to STRING (https://string-db.org/) to generate the PPI axis, and the results were visualized using the Cytoscape (v3.8.2) software.^[11]^ Individual node degree was computed using Cytoscape. Lastly, genes with > 15 degree were considered to be candidate hub genes.

### 2.6. Construction and analysis of the miRNA-mRNA network

To explore correlations between miRNA and mRNA in the key module, we retrieved miRNA information from the miRTarBase database, including, over 50,000 experimentally validated miRNA-target associations. The miRNA-mRNA modulatory network was visualized using Cytoscape.

### 2.7. DEGs screening

DEGs were identified between NF2-Vs and normal nerves using the R package “limma.”^[12]^ The threshold parameters were: false discovery rate < 0.05 and |log_2_(fold change) | ≥ 1.

### 2.8. Identification of drug candidates

To identify small molecular drugs that may effectively inhibit NF2-VSs occurrence and progression, we uploaded our list of up- and down-regulated genes into the connectivity map (CMap) database. CMap, a gene expression database, was employed to screen for functional associations among small molecular compounds, genes, and disease status.^[13]^ A correlation score was then obtained from the CMap database based on the DEG enrichment in the reference gene expression.

## 3. Results

### 3.1. Weighted gene co-expression network (WGCN) construction

**Figure 1.**
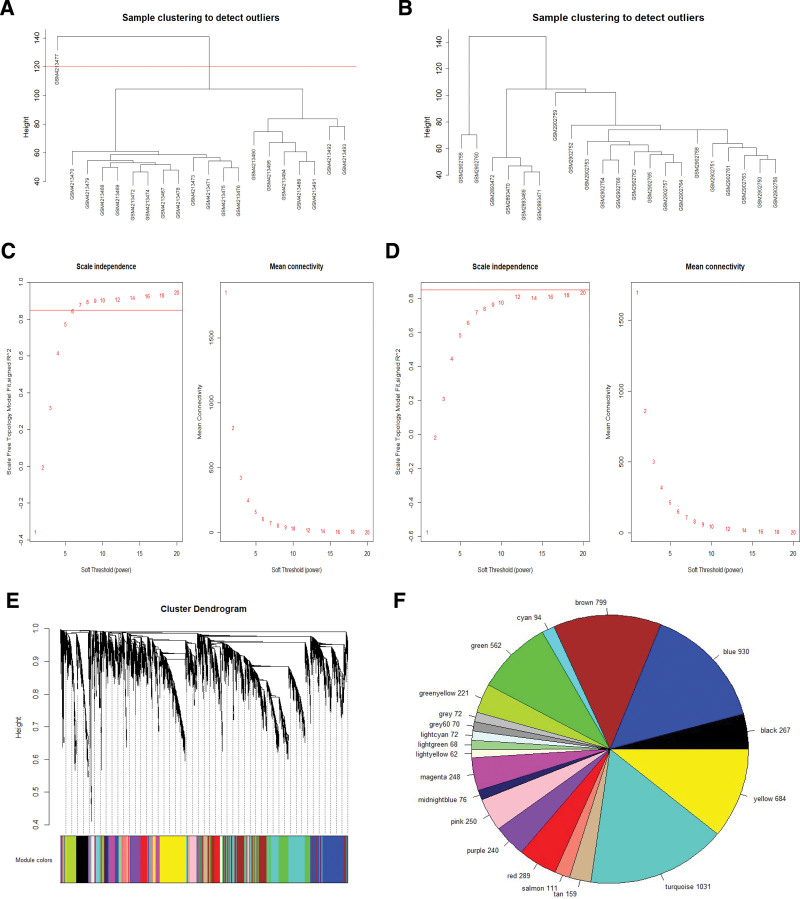
Construction of a co-expression network. (A) Sample clustering dendrogram of GSE141801 using WGCNA. (B) Sample clustering dendrogram of GSE108524 using WGCNA. (C) The scale-free fit index assessment for several soft-threshold powers from the GSE108524 dataset. Red line adjusted to 0.85. (D) The scale-free fit index assessment for several soft-threshold powers from the GSE141801 dataset. Red line adjusted to 0.85. (E) The cluster dendrogram of the co-expression network-based genes. (F) The gene quantities in different modules. WGCNA = weighted correlation network analysis.

The leading 25% of variant genes were entered in co-expression analysis. Sample hierarchical clustering methods were used to assess sample outliers in the GSE108524 and GSE141801 datasets, and GSM4213477 was eliminated from co-expression analysis (Fig. 1A and B). Next, the “pickSoftThreshold” tool from the WGCNA package was employed for soft-thresholding power (β) determination. The estimated GSE108524 power (β) was 7, however, no suitable power (β) was detected within the GSE141801 dataset (Fig. 1C and D). Thus, the WGCN was generated using 6305 genes from the GSE108524 dataset. Using WGCNA, 19 co-expression modules were identified (Fig. 1E and F), and the gray modules represented genes that were not classified into other modules.

### 3.2. Identification of module-trait associations and key modules screening

**Figure 2.**
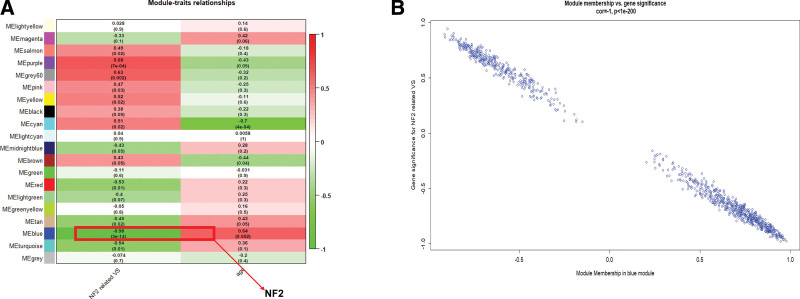
Screening of key modules. (A) Module-trait associations within the generated network. The top figure in each row denotes the relation to various clinical features, whereas, the bottom figure denotes the *P* value. The red box represents the module with NF2. (B) The NF2 gene relevance in the blue module (one dot refers to a single gene within the blue module). NF2 = neurofibromatosis type 2.

MEs were assessed for individual modules, and the association between MEs and NF2-VSs was computed. The associated heatmap is displayed in Figure 2A. We observed a strong negative correlation between the blue module and NF2-VSs (R = −0.98 and *P* < .001), as depicted in Figure 2B. Additionally, the NF2 gene was also located within the blue module (Fig. 2A). Hence, the blue module was selected as the key module. Based on our WGCNA, 215 candidate hub genes were finally screened from the blue module.

### 3.3. FEA of genes in the blue module

**Figure 3.**
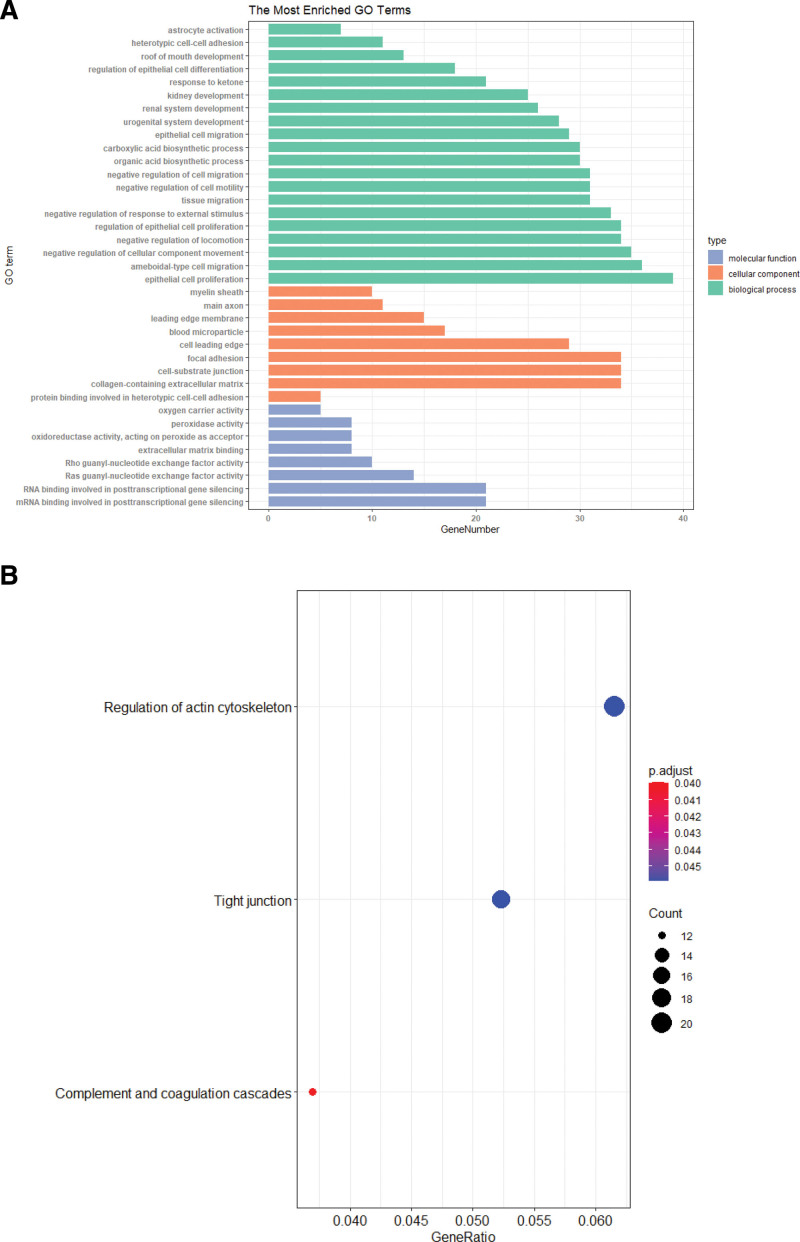
GO and KEGG network enrichment analyses of key module genes. (A) GO analysis of blue module genes. Only the top 20 BP terms are displayed. (B) KEGG network analysis of blue module genes. BP = biological process, GO = gene ontology, KEGG = Kyoto Encyclopedia of Genes and Genomes.

Gene ontology (GO) term and Kyoto Encyclopedia of Genes and Genomes (KEGG) FEA were conducted with the clusterProfiler package, and our conclusions are presented in Figure 3. The analyzed genes were strongly enriched in the following biological process terms, namely, cell proliferation, cell migration, and movement; as well as the molecular function terms “mRNA binding involved in posttranscriptional gene silencing” and “RNA binding involved in posttranscriptional gene silencing,” and cellular components related to the collagen-based extracellular matrix, cell-substrate junction, and focal adhesion (Fig. 3A). Moreover, the genes showed marked enrichment in 3 KEGG axes, namely, complement and coagulation cascades, tight junction, and actin cytoskeleton modulation (Fig. 3B).

### 3.4. PPI axis construction

**Figure 4.**
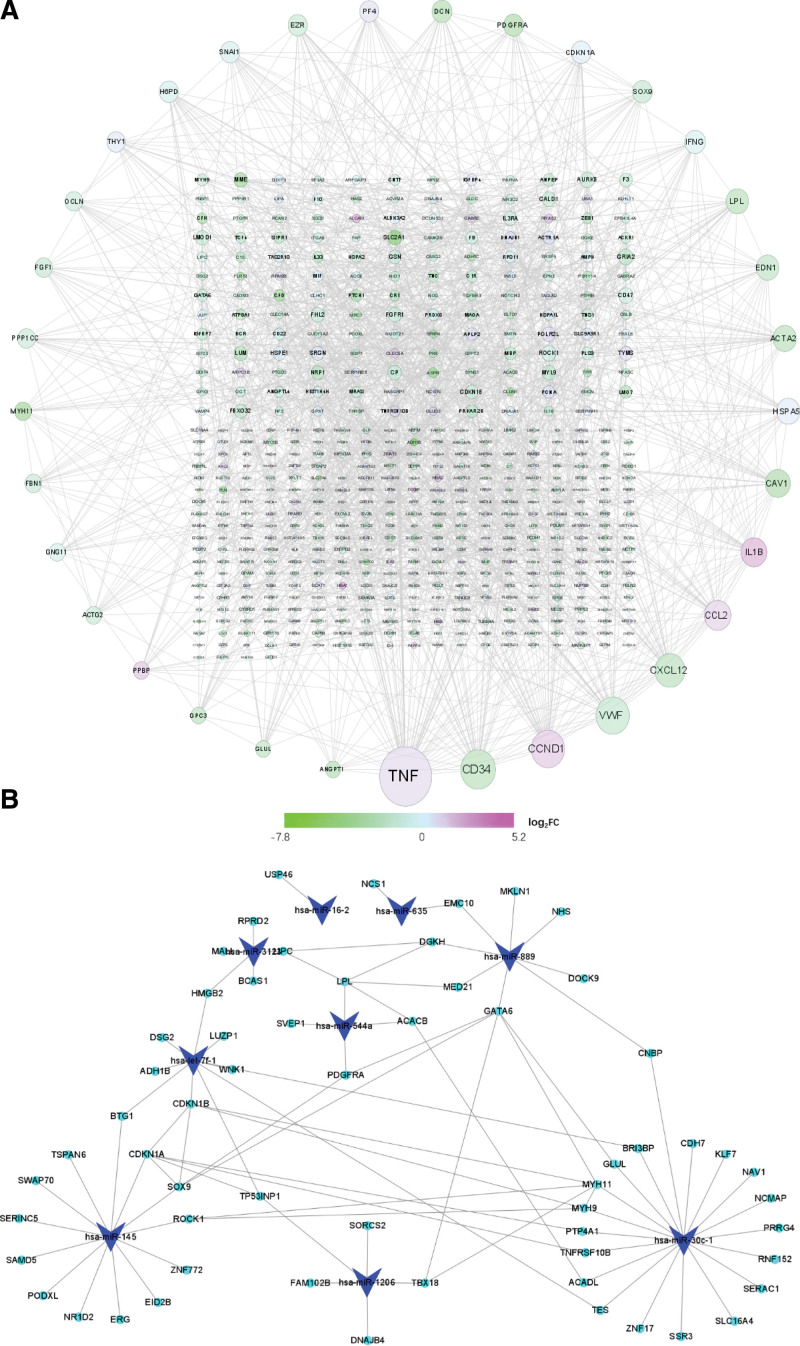
The blue module network. (A) PPI network of blue module genes, the node size indicates the network degree, the log_2_FC rang is presented as a color bar located at the bottom of the figure. The outer layer nodes represent genes with degrees > 15. (B) microRNA-target modulatory axis for the blue module. Blue triangles denote microRNAs, and green nodes denote genes. PPI = protein-protein interaction.

The STRING database was used to generate the PPI axis of genes in the blue module. PPIs with interaction scores > 0.4 was employed for PPI axis construction. In all, 33 genes (15.35% of the candidate hub genes) with degrees > 15 were recognized as candidate hub genes belonging to the PPI axis (Fig. 4A).

### 3.5. The mRNA-miRNA modulatory network construction

The blue module consisted of 90 microRNAs (41.86% of the candidate hub genes). We retrieved 11 microRNAs (12.22% of the microRNAs) with |MM| >0.8 as candidate hub microRNAs. Subsequently, we employed the miRTarBase database to predict the target genes of 11 miRNAs. In all, 1586 genes were estimated, which were matched to 60 mRNAs (27.91% of the candidate hub genes) from the blue module. The miRNA-mRNA modulatory axis is displayed in Figure 4B.

### 3.6. Identification of DEGs and real hub genes

**Figure 5.**
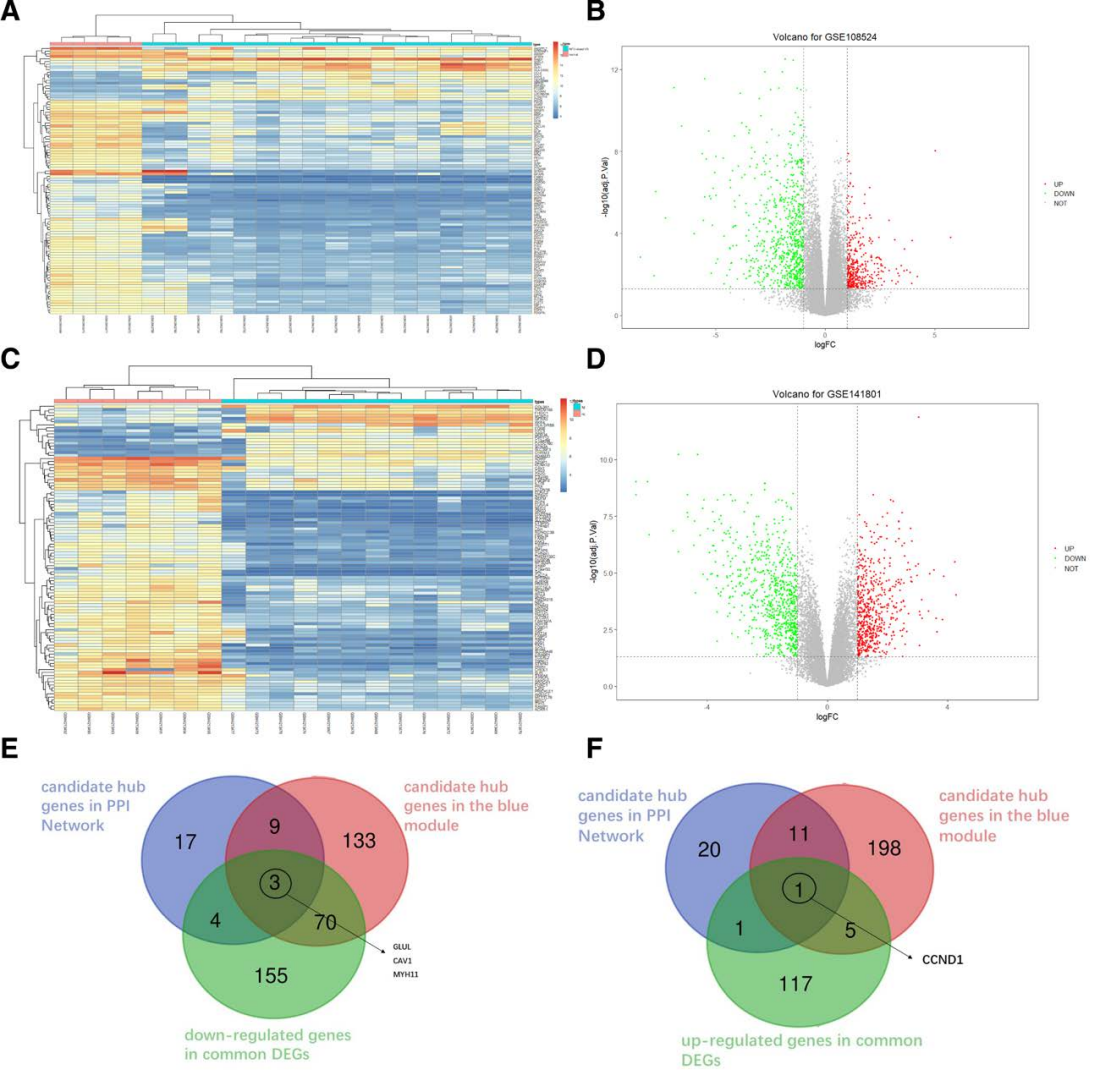
Identification of real hub genes. (A) Heatmap of top 100 DEGs from the GSE108524 dataset. (B) Volcano plot of DEGs in the GSE108524 dataset. (C) Heatmap of top 100 DEGs from the GSE141801 dataset. (D) Volcano plot of DEGs in the GSE141801 dataset. (E) Three genes were identified by intersecting the scarcely-expressed common DEGs, candidate hub genes from the PPI axis, and potential hub genes from the blue module. (F) One gene from the intersection of highly-expressed common DEGs, candidate hub genes from the PPI axis, and potential hub genes from the blue module. DEGs = differentially expressed genes, PPI = protein-protein interaction.

Following DEG screening based on the aforementioned criteria, 1124 and 1308 genes were identified from the GSE108524 and GSE141801 datasets, respectively. The top 100 DEGs for both datasets were showed in Figure 5A and C, respectively. These genes shared 356 DEGs (17.15% of total DEGs) between the 2 datasets, among which, 232 (65.17% of the shared DEGs) were scarcely expressed and 124 (34.83% of the shared DEGs) were highly expressed. The volcano plots for the DEGs of GSE108524 and GSE141801 datasets were presented in Figure 5B and D. Finally, 4 genes (1.12% of the shared DEGs) were screened as real hub genes upon intersection of the common DEGs, candidate hub genes from the PPI axis, and potential hub genes from the blue module (Fig. 5E and F).

### 3.7. Screening of potential therapeutic drugs

To explore drugs that potentially inhibit NF2-VSs development and progression, we converted the format of common elevated- and reduced gene expressions into probe IDs within the HG-U133A platform, and uploaded the information into the CMap database. Subsequently, negative enrichment score-based stratification was used to identify leading small molecular compounds for drug development. Drug candidates with enrichment scores < −0.7 are provided in Table 1.

**Table 1:** Drug candidates for NF2.

CMap.Name	PubChem CID	Enrichment	*P*
5279552	5279552	−0.959	.00368
Proscillaridin	5284613	−0.928	.00058
Naftopidil	4418	−0.874	.00403
Fursultiamine	3002119	−0.805	.0028
Cefalonium	21743	−0.797	.01711
Tolazamide	5503	−0.78	.02189
Ursolic acid	64945	−0.772	.00547
Clidinium bromide	19004	−0.75	.00776
Dropropizine	3169	−0.745	.00839
Dicloxacillin	18381	−0.704	.01581

CMap = connectivity map, NF2 = neurofibromatosis type 2.

## 4. Discussion

NF2 is an autosomal dominant inherited disease brought on by variants within the NF2 gene. The classical NF2 symptom is bilateral VSs^[14]^; the NF2 incidence at birth is approximately 1/33,000; and its overall prevalence is about 1/56,000.^[15]^ NF2, harboring 17 exons, is a tumor suppressor gene. It encodes a protein named merlin containing 559 amino acids.^[16]^ Merlin, belonging to the Ezrin/Radixin/Moesin family of membrane-cytoskeleton-interacting proteins, is primarily present within the plasma membrane and cytoskeletal compartments, and it interacts with multiple transmembrane receptors and intracellular proteins, namely, protocadherin fat (FAT), CD44, β1-integrin, and EGFR, thereby providing anchorage between the aforementioned proteins.^[17]^ Despite strong evidence that merlin contributes to the stability of the membrane-cytoskeleton interface by suppressing the PI3kinase/Akt, Raf/MEK/ERK, and mTOR networks,^[18–20]^ not much is known about the NF2 tumorigenesis process.^[21]^ At present, bioinformatics analyses are used to elucidate the underlying mechanisms and potential therapeutic agents of numerous tumors. Herein, using WGCNA, we generated the NF2-based gene co-expression axes, and identified a key gene co-expression module strongly associated with NF2-VS pathogenesis. We also identified several drugs with potential therapeutic benefits for NF2-VSs using the CMap database. Based on our literature review, this study was the first to employ bioinformatics analysis to screen for hub genes and drug candidates for NF2-VSs.

Herein, we identified 19 co-expression modules using WGCNA. NF2-VS was most significantly correlated with the blue module. Moreover, since the NF2 gene was detected within the blue module, this module was then selected as the primary module.

GO and KEGG FEA are commonly used to elucidate the physiological roles of relevant genes. Herein, we employed the GO and KEGG analyses to examine the physiological activities of genes within the blue module. Based on the GO analysis, significant enrichments were found in cell proliferation and migration (in biological process), as well as cell adhesion and junction (in cellular components). Based on the KEGG network analyses, the actin cytoskeleton modulation was intricately related to NF2, which corroborated with the conclusions of prior studies.

In our attempt to identify real hub genes, we identified 356 common DEGs between NF2-VSs and normal nerves from the GSE108524 and GSE141801 datasets. We also employed STRING to construct the PPI axis of genes from the blue module. Ultimately, we identified 4 genes (GLUL, CAV1, MYH11, CCND1) as real hub genes via the intersection of common DEGs, genes with degree > 15 from the PPI axis, and potential hub genes from the blue module. GLUL encodes glutamine synthetase, the only enzyme that catalyzes the novel production of glutamine via condensation of ammonium and glutamate into glutamine.^[22]^ GLUL is involved in several cancers.^[23–25]^ Moreover, GLUL knockdown is reported to abrogate membranal localization and impair endothelial cell motility within human umbilical vein endothelial cells.^[26]^ CAV1 is a membranal protein that coats the plasma membrane caveolae.^[27]^ CAV1 phosphorylation is strongly associated with cellular transformation.^[28,29]^ Prior investigations revealed that CAV1 reduces xenograft tumor formation from colon cancer cells within nude mice.^[30]^ CAV1 content is known to positively regulate patient prognosis in various tumor types.^[31–33]^ Stromal CAV1 deficiency is also reportedly linked to autophagy, hypoxia, and oxidative stress.^[32–35]^ MYH encodes myosin heavy chain 11, a smooth muscle myosin that belongs to the myosin heavy chain family.^[36]^ In recent years, a series of reports indicated that MYH11 modulates cell migration, association with cell adhesion proteins, and tumor suppression.^[37,38]^ Moreover, mutations in the MYH11 gene is related to multiple cancer types, namely, colorectal, bladder,^[39]^ and head and neck cancers.^[40]^ Herein, CCND1 was the only hub gene that was elevated in NF2-VS samples. CCND1 encodes cyclin D1, a major cell cycle regulator.^[41]^ Cyclin proteins D1 control cell cycle progression via modulating the G1-S phase transition.^[42]^ CCND1 is an oncogene responsible for uncontrolled cell proliferation.^[43]^ More importantly, merlin is known to inhibit CCND1.^[44]^

Based on earlier reports, miRNAs are critical regulators of numerous tumors. Therefore, we selected miRNAs with high |MM| to generate a potential miRNA-targeted modulatory network. According to our analysis, miR-30c-1 (degree = 19) and miR-145 (degree = 13) are potential modulators of NF2-VSs.

We also identified candidate therapeutic drugs that may target common highly- and scarcely-regulated genes in NF2-VSs using the CMap database (Table 1). Proscillaridin,^[45]^ naftopidil,^[46]^ fursultiamine, and ursolic acid^[47]^ are reported to possess robust antitumor activity. Among them, proscillaridin yields a higher enrichment score (enrichment = −0.928, *P* = .00058). Proscillaridin, a cardiac glycoside, is derived from Scilla and in *Drimia maritima* plants, and it possesses potent cytotoxic and anticancer properties. Collectively, our analyses indicated that these candidate drugs may be effectively employed to manage NF2-VSs.

## 5. Conclusions

In conclusion, herein, we demonstrated that the key gene co-expression module, hub genes, and several functional networks, such as, actin cytoskeleton regulation, cell proliferation, and migration were strongly associated with NF2 pathogenesis. We also identified potential drugs that may inhibit NF2-VSs. These results are crucial for the future development and management of NF2-VSs. We warrant additional investigations into the underlying mechanism of hub genes and functional networks that potentially contributes to NF2 development.

## Author contributions

**Conceptualization:** Jiasheng Yuan, Yanpeng Fu.

**Data curation:** Jiasheng Yuan, Yanpeng Fu.

**Formal analysis:** Jiasheng Yuan, Yanpeng Fu.

**Funding acquisition:** Jiasheng Yuan, Yanpeng Fu.

**Investigation:** Jiasheng Yuan, Yanpeng Fu.

**Methodology:** Jiasheng Yuan, Yanpeng Fu.

**Project administration:** Yuehui Liu.

**Resources:** Jiasheng Yuan, Yanpeng Fu.

**Software:** Jiasheng Yuan, Yanpeng Fu.

**Supervision:** Yuehui Liu.

**Validation:** Yuehui Liu.

**Visualization:** Jiasheng Yuan, Yanpeng Fu.

**Writing – original draft:** Jiasheng Yuan, Yanpeng Fu.

**Writing – review & editing:** Jiasheng Yuan, Yanpeng Fu, Yuehui Liu.

## References

[1] Stemmer-Rachamimov AO Louis DN Nielsen GP. Comparative pathology of nerve sheath tumors in mouse models and humans. Cancer Res. 2004;64:3718–24. 15150133 10.1158/0008-5472.CAN-03-4079

[2] Louis DN Perry A Reifenberger G. The 2016 World Health Organization classification of tumors of the central nervous system: a summary. Acta Neuropathol. 2016;131:803–20. 27157931 10.1007/s00401-016-1545-1

[3] Evans DG Moran A King A. Incidence of vestibular schwannoma and neurofibromatosis 2 in the North West of England over a 10-year period: higher incidence than previously thought. Otol Neurotol. 2005;26:93–7. 15699726 10.1097/00129492-200501000-00016

[4] Halliday J Rutherford SA McCabe MG. An update on the diagnosis and treatment of vestibular schwannoma. Expert Rev Neurother. 2018;18:29–39. 29088993 10.1080/14737175.2018.1399795

[5] den Bakker MA Vissers KJ Molijn AC. Expression of the neurofibromatosis type 2 gene in human tissues. J Histochem Cytochem. 1999;47:1471–80. 10544220 10.1177/002215549904701113

[6] Morrison H Sherman LS Legg J. The NF2 tumor suppressor gene product, merlin, mediates contact inhibition of growth through interactions with CD44. Genes Dev. 2001;15:968–80. 11316791 10.1101/gad.189601 PMC312675

[7] Martinez-Glez V Franco-Hernandez C Rey JA. Microarray gene expression profiling in meningiomas and schwannomas. Curr Med Chem. 2008;15:826–33. 18393851 10.2174/092986708783955527

[8] Barrett T Wilhite SE Ledoux P. NCBI GEO: archive for functional genomics data sets--update. Nucleic Acids Res. 2013;41:D991–5. 23193258 10.1093/nar/gks1193 PMC3531084

[9] Langfelder P Horvath S. WGCNA: an R package for weighted correlation network analysis. BMC Bioinf. 2008;9:559. 10.1186/1471-2105-9-559 PMC2631488 19114008

[10] Yu G Wang LG Han Y. clusterProfiler: an R package for comparing biological themes among gene clusters. OMICS. 2012;16:284–7. 22455463 10.1089/omi.2011.0118 PMC3339379

[11] Shannon P Markiel A Ozier O. Cytoscape: a software environment for integrated models of biomolecular interaction networks. Genome Res. 2003;13:2498–504. 14597658 10.1101/gr.1239303 PMC403769

[12] Ritchie ME Phipson B Wu D. limma powers differential expression analyses for RNA-sequencing and microarray studies. Nucleic Acids Res. 2015;43:e47. 25605792 10.1093/nar/gkv007 PMC4402510

[13] Lamb J Crawford ED Peck D. The connectivity map: using gene-expression signatures to connect small molecules, genes, and disease. Science. 2006;313:1929–35. 17008526 10.1126/science.1132939

[14] Howitz MF Johansen C Tos M. Incidence of vestibular schwannoma in Denmark, 1977–1995. Am J Otol. 2000;21:690–4. 10993460

[15] Evans DG Howard E Giblin C. Birth incidence and prevalence of tumor-prone syndromes: estimates from a UK family genetic register service. Am J Med Genet A. 2010;152A:327–32. 20082463 10.1002/ajmg.a.33139

[16] Pecina-Slaus N. Merlin, the NF2 gene product. Pathol Oncol Res. 2013;19:365–73. 23666797 10.1007/s12253-013-9644-y

[17] Stamenkovic I Yu Q. Merlin, a “magic” linker between extracellular cues and intracellular signaling pathways that regulate cell motility, proliferation, and survival. Curr Protein Pept Sci. 2010;11:471–84. 20491622 10.2174/138920310791824011 PMC2946555

[18] Rong R Tang X Gutmann DH. Neurofibromatosis 2 (NF2) tumor suppressor merlin inhibits phosphatidylinositol 3-kinase through binding to PIKE-L. Proc Natl Acad Sci U S A. 2004;101:18200–5. 15598747 10.1073/pnas.0405971102 PMC535703

[19] Li W You L Cooper J. Merlin/NF2 suppresses tumorigenesis by inhibiting the E3 ubiquitin ligase CRL4(DCAF1) in the nucleus. Cell. 2010;140:477–90. 20178741 10.1016/j.cell.2010.01.029 PMC2828953

[20] James MF Stivison E Beauchamp R. Regulation of mTOR complex 2 signaling in neurofibromatosis 2-deficient target cell types. Mol Cancer Res. 2012;10:649–59. 22426462 10.1158/1541-7786.MCR-11-0425-T

[21] Kim D Song J Lee S. An integrative transcriptomic analysis of systemic juvenile idiopathic arthritis for identifying potential genetic markers and drug candidates. Int J Mol Sci. 2021;22:712. 33445803 10.3390/ijms22020712 PMC7828236

[22] Bott AJ Shen J Tonelli C. Glutamine anabolism plays a critical role in pancreatic cancer by coupling carbon and nitrogen metabolism. Cell Rep. 2019;29:1287–1298.e6. 31665640 10.1016/j.celrep.2019.09.056 PMC6886125

[23] Bott AJ Peng IC Fan Y. Oncogenic Myc induces expression of glutamine synthetase through promoter demethylation. Cell Metab. 2015;22:1068–77. 26603296 10.1016/j.cmet.2015.09.025 PMC4670565

[24] Cox AG Hwang KL Brown KK. Yap reprograms glutamine metabolism to increase nucleotide biosynthesis and enable liver growth. Nat Cell Biol. 2016;18:886–96. 27428308 10.1038/ncb3389 PMC4990146

[25] Tardito S Oudin A Ahmed SU. Glutamine synthetase activity fuels nucleotide biosynthesis and supports growth of glutamine-restricted glioblastoma. Nat Cell Biol. 2015;17:1556–68. 26595383 10.1038/ncb3272 PMC4663685

[26] Eelen G Dubois C Cantelmo AR. Role of glutamine synthetase in angiogenesis beyond glutamine synthesis. Nature. 2018;561:63–9. 30158707 10.1038/s41586-018-0466-7

[27] Parton RG. Caveolae: structure, function, and relationship to disease. Annu Rev Cell Dev Biol. 2018;34:111–36. 30296391 10.1146/annurev-cellbio-100617-062737

[28] Glenney JJ Zokas L. Novel tyrosine kinase substrates from Rous sarcoma virus-transformed cells are present in the membrane skeleton. J Cell Biol. 1989;108:2401–8. 2472406 10.1083/jcb.108.6.2401 PMC2115592

[29] Li S Seitz R Lisanti MP. Phosphorylation of caveolin by Src tyrosine kinases the alpha-isoform of caveolin is selectively phosphorylated by v-Src in vivo. J Biol Chem. 1996;271:3863–8. 8632005

[30] Bender FC Reymond MA Bron C. Caveolin-1 levels are down-regulated in human colon tumors, and ectopic expression of caveolin-1 in colon carcinoma cell lines reduces cell tumorigenicity. Cancer Res. 2000;60:5870–8. 11059785

[31] Williams TM Lisanti MP. Caveolin-1 in oncogenic transformation, cancer, and metastasis. Am J Physiol Cell Physiol. 2005;288:C494–506. 15692148 10.1152/ajpcell.00458.2004

[32] Goetz JG Lajoie P Wiseman SM. Caveolin-1 in tumor progression: the good, the bad and the ugly. Cancer Metastasis Rev. 2008;27:715–35. 18506396 10.1007/s10555-008-9160-9

[33] Nunez-Wehinger S Ortiz RJ Diaz N. Caveolin-1 in cell migration and metastasis. Curr Mol Med. 2014;14:255–74. 24467203 10.2174/1566524014666140128112827

[34] Sotgia F Martinez-Outschoorn UE Pavlides S. Understanding the Warburg effect and the prognostic value of stromal caveolin-1 as a marker of a lethal tumor microenvironment. Breast Cancer Res. 2011;13:213. 21867571 10.1186/bcr2892 PMC3236330

[35] Witkiewicz AK Dasgupta A Sotgia F. An absence of stromal caveolin-1 expression predicts early tumor recurrence and poor clinical outcome in human breast cancers. Am J Pathol. 2009;174:2023–34. 19411448 10.2353/ajpath.2009.080873 PMC2684168

[36] Matsuoka R Yoshida MC Furutani Y. Human smooth muscle myosin heavy chain gene mapped to chromosomal region 16q12. Am J Med Genet. 1993;46:61–7. 7684189 10.1002/ajmg.1320460110

[37] Xi WD Liu YJ Sun XB. Bioinformatics analysis of RNA-seq data revealed critical genes in colon adenocarcinoma. Eur Rev Med Pharmacol Sci. 2017;21:3012–20. 28742206

[38] Wang RJ Wu P Cai GX. Down-regulated MYH11 expression correlates with poor prognosis in stage II and III colorectal cancer. Asian Pac J Cancer Prev. 2014;15:7223–8. 25227818 10.7314/apjcp.2014.15.17.7223

[39] Hu J Zhou L Song Z. The identification of new biomarkers for bladder cancer: a study based on TCGA and GEO datasets. J Cell Physiol. 2019;234:15607–18. 30779109 10.1002/jcp.28208

[40] Islam T Rahman R Gov E. Drug targeting and biomarkers in head and neck cancers: insights from systems biology analyses. OMICS. 2018;22:422–36. 29927717 10.1089/omi.2018.0048

[41] Gonzalez-Ruiz L Gonzalez-Moles MA Gonzalez-Ruiz I. An update on the implications of cyclin D1 in melanomas. Pigment Cell Melanoma Res. 2020;33:788–805. 32147907 10.1111/pcmr.12874

[42] Malumbres M Barbacid M. Cell cycle, CDKs and cancer: a changing paradigm. Nat Rev Cancer. 2009;9:153–66. 19238148 10.1038/nrc2602

[43] Hanahan D Weinberg RA. The hallmarks of cancer. Cell. 2000;100:57–70. 10647931 10.1016/s0092-8674(00)81683-9

[44] Xiao GH Gallagher R Shetler J. The *NF2* tumor suppressor gene product, merlin, inhibits cell proliferation and cell cycle progression by repressing cyclin D1 expression. Mol Cell Biol. 2005;25:2384–94. 15743831 10.1128/MCB.25.6.2384-2394.2005 PMC1061616

[45] Berges R Denicolai E Tchoghandjian A. Proscillaridin A exerts anti-tumor effects through GSK3beta activation and alteration of microtubule dynamics in glioblastoma. Cell Death Dis. 2018;9:984. 30250248 10.1038/s41419-018-1018-7 PMC6155148

[46] Hori Y Ishii K Kanda H. Naftopidil, a selective alpha1-adrenoceptor antagonist, suppresses human prostate tumor growth by altering interactions between tumor cells and stroma. Cancer Prev Res (Phila). 2011;4:87–96. 21205739 10.1158/1940-6207.CAPR-10-0189

[47] Zhang N Liu S Shi S. Solubilization and delivery of Ursolic-acid for modulating tumor microenvironment and regulatory T cell activities in cancer immunotherapy. J Control Release. 2020;320:168–78. 31926193 10.1016/j.jconrel.2020.01.015

